# Utilization of Alternative Fibres Manufactured from Recycled PET Bottles in Concrete Technology for the Improvement of Fire Resistance

**DOI:** 10.3390/polym16223145

**Published:** 2024-11-12

**Authors:** Martin Sedlmajer, Jiří Zach, Jan Bubeník, Jiří Bydžovský, Vítězslav Novák

**Affiliations:** Faculty of Civil Engineering, Brno University of Technology, 612 00 Brno, Czech Republic; jiri.zach@vut.cz (J.Z.); jan.bubenik@vut.cz (J.B.); jiri.bydzovsky@vut.cz (J.B.); vitezslav.novak1@vut.cz (V.N.)

**Keywords:** PET—polyethylene terephthalate, polymer fibres, recycling, PET bottles, concrete, fire resistance

## Abstract

This article addresses the potential use of secondary polymer fibres in the field of structural concrete as a replacement for primary polymer fibres (mainly polypropylene/PP/), which are used in concrete to enhance its resistance when exposed to high temperatures (especially in the case of fire). Research has shown that, in addition to PP fibres, polyethylene terephthalate/PET/fibres, produced by recycling packaging materials (mainly PET bottles), can also be used as an alternative. These fibres are industrially produced in similar dimensions as PP fibres and exhibit similar behaviour when added to fresh and hardened concrete. In terms of their effect on increasing resistance to extreme heat loads, it has been found that despite a higher melting point (Tm), concrete with these fibres demonstrates comparable fire resistance. Therefore, it can be concluded that secondary PET fibres represent an interesting alternative to primary PP fibres from the perspective of a circular economy, and their use in construction represents a potentially valuable application for PET obtained through the collection and recycling of PET packaging materials.

## 1. Introduction

Plastics are materials that are characterized by low cost, weight, high resistance, and long-term durability. For these reasons, plastics are being used in increasing quantities. Technologically, it is a unique way of technological solutions, production, or production in many industries. Plastic materials have helped in many cases and industries. In some cases, it is very difficult to do without them.

### 1.1. Recycling of Packaging/Plastics in General, Circular Economy

**Figure 1:**
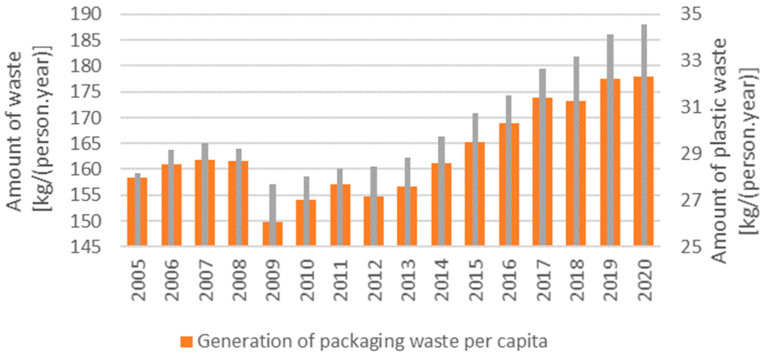
Generation of packaging waste (orange column) and plastic packaging waste (grey column) per capita [4].

In 1950, approximately 1.5 million tons of plastic were produced around the world, and in 2018, the amount of plastic produced reached 359 million tons. Plastics production continues to grow, and it is projected that by 2050, total production will reach 700 million tons. The growing amount of plastic in the global circulation has resulted in a significant ecological burden. In the EU, approximately 40% of the plastics produced are used for short-term applications, such as packaging plastics (bags, bottles, wrapping films) [1]. Therefore, effective recycling of these materials is emerging as a key issue in environmental sustainability [2,3,4]. The EU aims to ensure that, by 2030, all packaging on its market is reusable or recyclable. The amount of packaging waste has increased slightly in recent years, with plastic packaging accounting for a significant portion, around 19% by weight, as shown in Figure 1. Due to the very low weight of plastic packaging waste, the volume of plastic packaging waste is proportionally much higher than that of other types of packaging.

**Figure 2:**
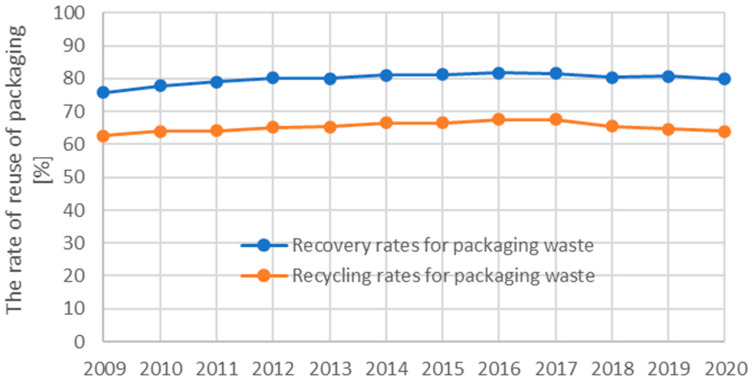
Recovery and recycling rates for packaging waste [4].

The production of packaging waste is also dependent on the rate of reuse of packaging materials within the EU, which has stagnated in recent years, see Figure 2.

From an environmental perspective, plastics represent a group of products that are highly energy- and resource-intensive. When recalculated per unit of volume, its recycling at the end of its useful life is very important, especially in light of the new European Green Deal [5] and other environmental regulations and requirements that are in force in most developed countries around the world. However, for polymer materials, their actual environmental impact is lower when calculated on the basis of the real mass embedded in kilograms in various applications, as polymer materials can be used in relatively thin layers, reducing their negative impact. However, this fact often complicates its recycling after the end of its useful life, especially when it is applied to the surface of other materials or when different types of plastic are combined in products, making it difficult to effectively separate them. One of the main producers of waste is the packaging industry, where efforts are being made to recycle individual packaging directly in a closed loop through targeted separation, using the “Bottle-to-Bottle Recycling” method. As numerous studies show, up to 75% of secondary packaging can be reused in the production of new packaging [6]. Despite these positive developments, the real situation is significantly more complex, and the lifecycle and recycling of secondary polymers (most commonly PET in the packaging industry) must consider all processes, from the logistical transportation of PET bottles to recycling centres through separation, sorting, and cleaning of the bottles to the process of preparing secondary raw material and transporting it to the manufacturer. Despite the current efforts in EU countries to prefer circular economy policies and to process waste where it is generated, it is certainly potentially interesting to explore other uses for secondary polymer substances in other industries, such as construction and the use of polymer fibres in concrete [7]. The production of polymer fibres from recycled plastics is also interesting due to the relatively low energy demand of the recycling process and the lower requirement for the purity of the input material compared to the use of recycled materials in the packaging industry [8]. This article focuses on the utilization of recycled PET bottles.

### 1.2. Recycling of PET Bottles and Production of PET Fibres

PET recycling is carried out in two ways: mechanical (84% of recycling) and chemical (16% of recycling). Mechanical recycling is most commonly performed via extrusion, which uses the principle of increased temperature and a rotating screw to induce softening or plastification [9]. The material is then passed through rollers with controlled temperature to produce a solid extrudate [10]. The principle of mechanical recycling of PET involves its degradation by cutting the chains, which leads to a reduction in the molecular weight of the polymer (potential by-products, such as carbon dioxide, water, and others are formed). Shortened chains reduce the elasticity of the polymer, reduce its viscosity, and make it brittle. PET chains break down due to the action of free radicals or carbon-to-hydrogen transfer [11].

Chemical recycling focuses on breaking down PET chains into basic monomers (depolymerization). The breakdown occurs by solvolysis or pyrolysis (thermal degradation in the absence of oxygen, air, or vacuum). This depolymerized PET waste is followed by the regeneration and refinement of the resulting monomers (or oligomers) or their transformation into new products. This means that after the regeneration of monomers or oligomers, the product undergoes purification to a quality acceptable for reproducing PET materials or converting them into new products [12].

After mechanical recycling of the waste PET material, solid extrudate in the form of PET flakes is produced. These flakes can be processed in two ways. The first method involves reinserting them into an extruder, where they are melted and subsequently drawn into monofilaments, which are then cut to the desired lengths. The second method involves mechanically cutting the PET flakes into individual fibres [13,14].

### 1.3. The Use of Fibres in Concrete, Types of Fibres, and Their Chemistry and Behavior

A major drawback of all cement composites is volumetric changes that are accompanied by the formation of microcracks in the cement matrix. The use of polymer fibres helps regulate crack formation, especially autogenous shrinkage and drying shrinkage. When cracks form, tensile stress begins to transfer to the fibres that run across the cracks. Research describes the achievement of this effect with various types of fibres from a material base perspective [15,16,17,18].

Some studies describe the use of PET fibres obtained from recycled material and the benefits of improved performance, particularly stiffness and reduction in crack formation in cement composite [19]. However, these were macrofibres obtained by cutting PET bottles [20,21] or large strips from PET bottles [22]. The use of recycled PET as a replacement for aggregate was also investigated [23,24]. The anisotropy of PET and the good adhesion of recycled PET to the cement matrix were also verified [25]. The application of recycled PET is also evident in the form of nanofibres for cement-based materials, as confirmed by a study by Chinchillas-Chinchillas et al. [26], who verified the combination of PET nanofibres with polyacrylonitrile (PAN). These nanofibres reach a diameter of around 400 nm and increase compressive strength and flexural tensile strength by 26% and 89% while reducing drying shrinkage by up to 93% compared to the reference mix without reinforcement. These results have shown that recycled nanomaterials can have an exceptionally good application in cementitious materials.

Mand Kamal Askar et al. conducted a study on the use of polyethylene terephthalate (PET) in concrete and concluded that the use of PET fibres in concrete is one way to ecologically utilize PET at the end of its useful life in primary applications. The use of PET fibres can improve the mechanical properties of concrete under certain conditions, but it can also lead to negative effects on the properties of concrete in both fresh and hardened states. In general, they found that there is very little information on the properties and use of PET fibres in concrete, and this area requires further research [27].

### 1.4. The Use of Fibres in Concrete to Improve Resistance to High Temperatures

Polymer fibres have long been used to enhance the fire resistance of composite materials, including concrete. Polypropylene (PP) fibres are most commonly used for this purpose and represent a highly significant group of materials for dispersed reinforcement. Adding PP fibres to concrete increases its porosity and permeability during a fire. High pore pressure forms under high temperatures during a fire, exceeding the tensile strength of the concrete, which is the main cause of explosive spalling. At temperatures above 170 °C, PP fibres melt. After these fibres burn out, a densely interconnected space is formed where the increasing steam pressure can expand or even escape from the concrete towards a free edge. These processes are well described in the literature [28,29,30]. Kalifa et al. describe a clear difference between fibre-free concrete and concrete with PP fibres as the temperature increases, noting differences in crack distribution and thickness. The positive effect of PP fibres is presented at increasing temperatures [31]. The positive influence on fire resistance, specifically due to the effect of PP fibres, has also been described by other researchers [30,32,33,34,35,36,37].

Based on the conclusions presented, it can be inferred that using thinner and longer fibres is more suitable than increasing the dose of standard fibres. In terms of the specific type of polymer, it is essential that the polymer fibre melts at a temperature at which the capillary pressure from the physically and chemically bound water begins to rise. The partial pressure of released water vapour increases nonlinearly with temperature. First, free physically bound water is released from the material’s pore system, depending on the pore size, and then, at higher temperatures, chemically bound water starts to release. However, during a fire, the structure is subjected to one-sided loading, causing gradual moisture redistribution in the structure and increasing pressure in the pore system. As noted in several studies [38], capillary pressure begins to increase significantly from 100 °C. Therefore, polymer fibres must be active (as scientific studies indicate) in the range of 100 to 200 °C. However, it always depends on the type of composite, and in composites with higher porosity, fibres with a higher melting point, such as PET fibres, which typically have a melting point in the range of 220 to 240 °C, could also be effective.

## 2. Materials and Methods

The experimental part of the work was divided into two sections. The first section of the experiments focused on the characterization of polymer fibres and the study of their behaviour at elevated temperatures. The second section was dedicated to the behaviour of fibres in concrete.

In the first part of the work, four types of polymers were selected:

- Low-density polyethylene (LD-PE)—prepared in the lab from foil sheets.
- Polypropylene (PP)—from Chryso Chemie s.r.o., Prague, Czech Republic.
- Recycled polyethylene–terephthalate (RE-PET)—from CIUR a.s., Prague, Czech Republic.
- Polyester (PES)—from Cetex Institut GmbH, Chemnitz, Germany.

**Figure 3:**
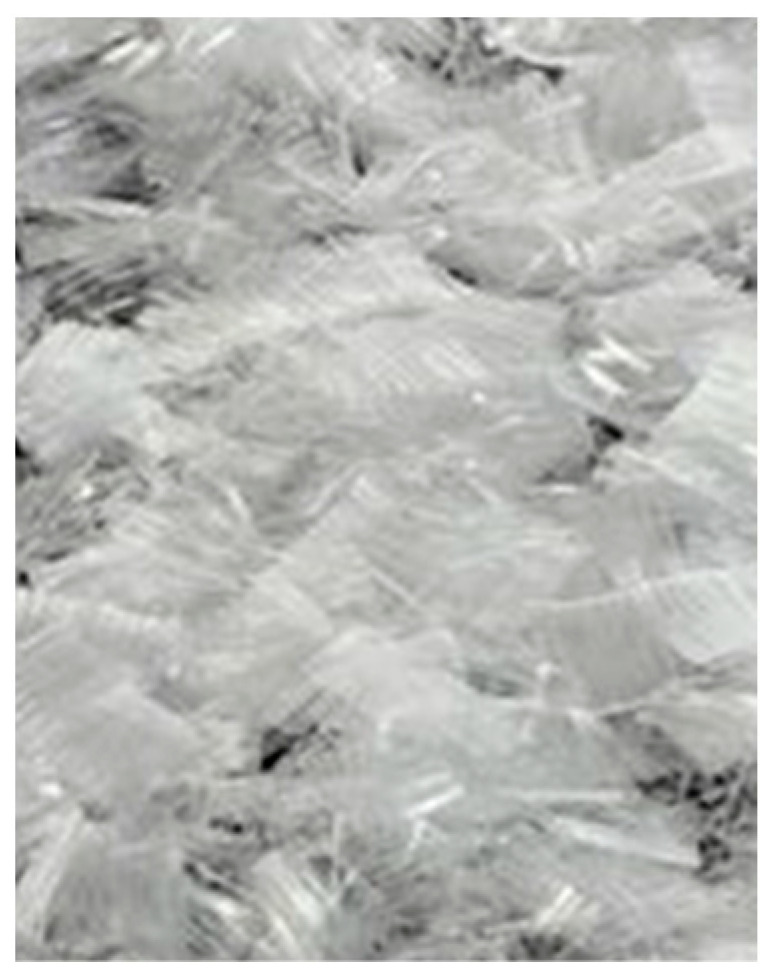
PP.

**Figure 4:**
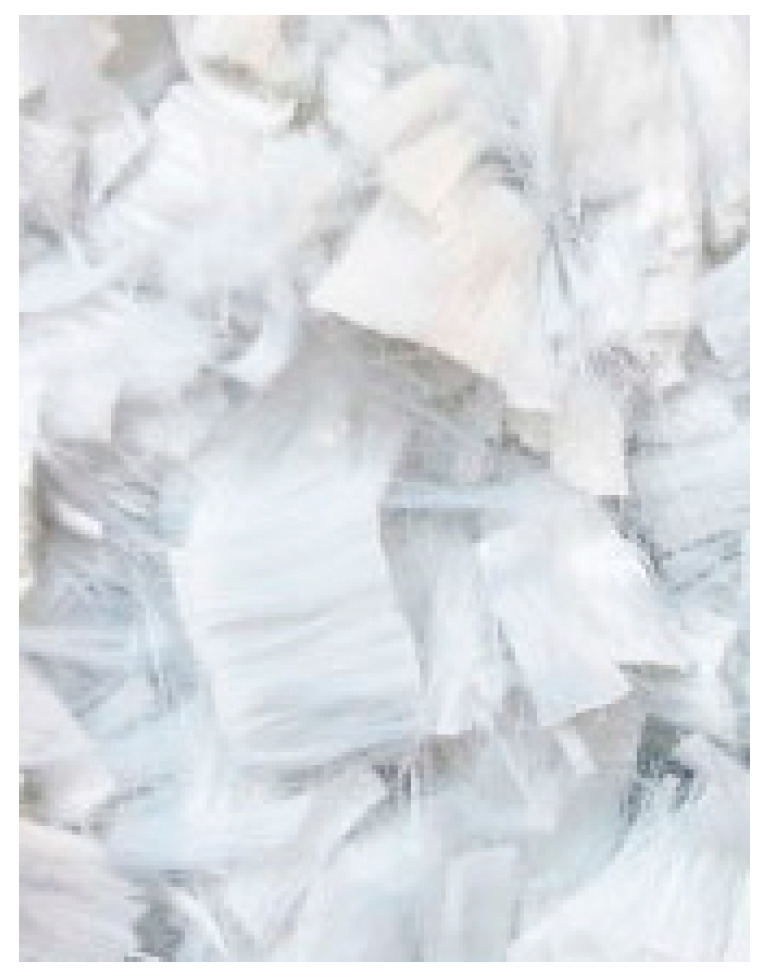
RE-PET.

**Figure 5:**
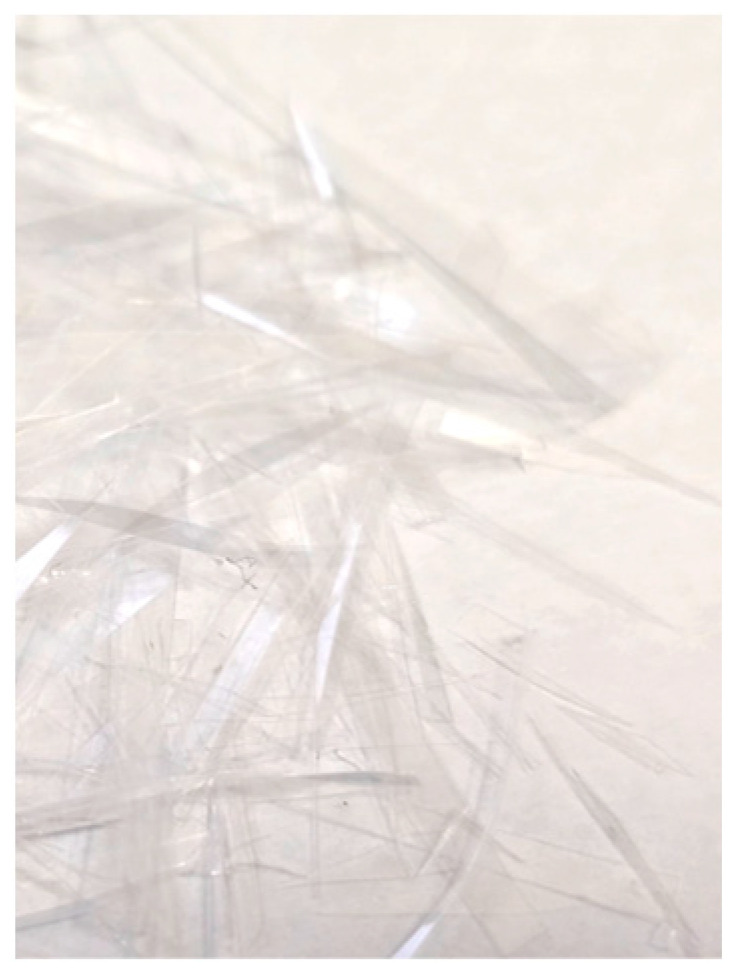
PE-LD.

**Figure 6:**
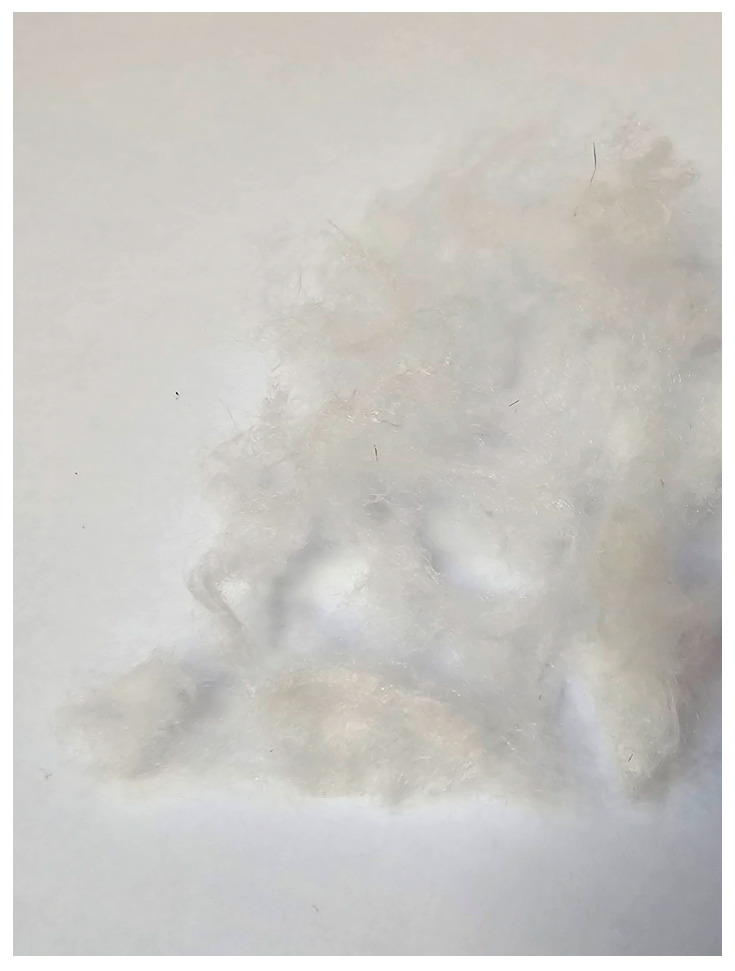
PES.

In the case of PP, RE-PET, and PES, these were fibres that could be added to cement composites. PE-LD was selected for comparison purposes, and the fibres were made from LD-PE film by cutting it into strips. The fibres used in the experiment can be seen in Figure 3, Figure 4, Figure 5 and Figure 6.

The second part of the work focused on studying the behaviour of selected polymer fibres in concrete, their impact on strength characteristics, consistency, and increased resistance to high temperatures.

### 2.1. Properties Tested on Polymer Materials

- Determination of basic properties (length, thickness) on an optical microscope.
- Study of the microstructure of fibres with the use of SEM.
- Study of behaviour under elevating temperature by TDA, DSC, and TG thermo analyze.
- Determination of combustion heat by calorimetry.

#### 2.1.1. Determination of Properties on an Optics Microscope

**Figure 7:**
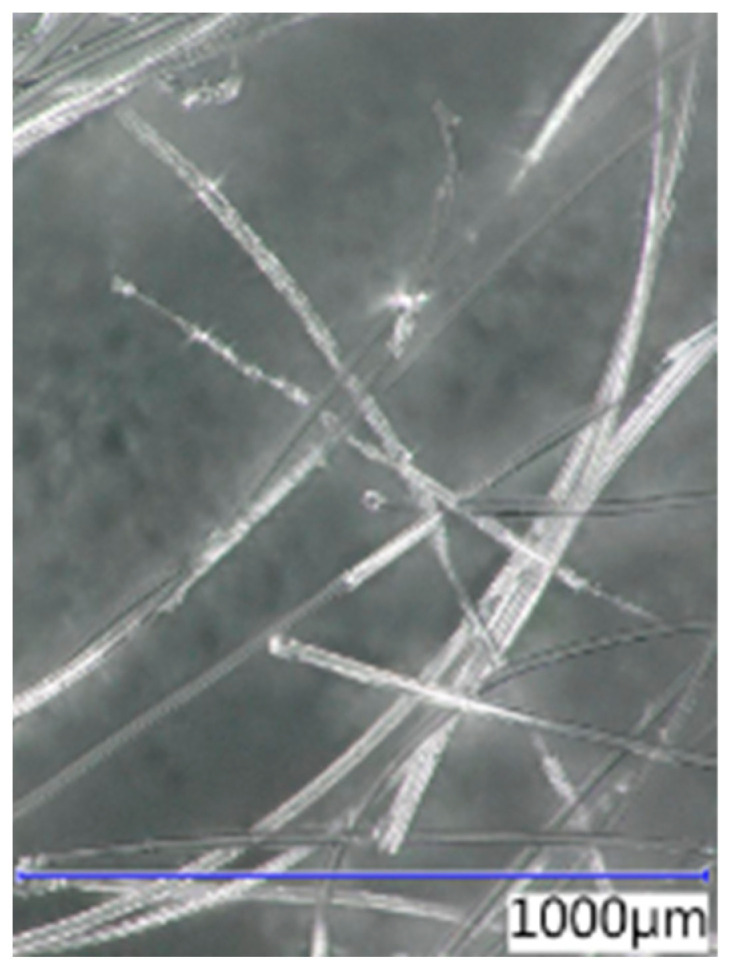
PP 65×.

**Figure 8:**
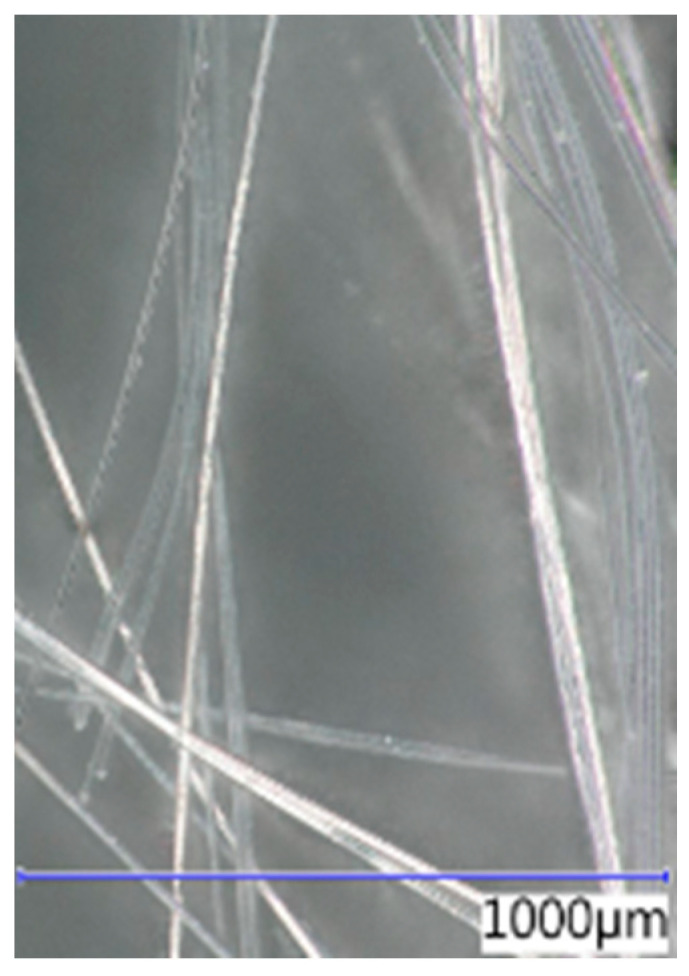
RE-PET 65×.

**Figure 9:**
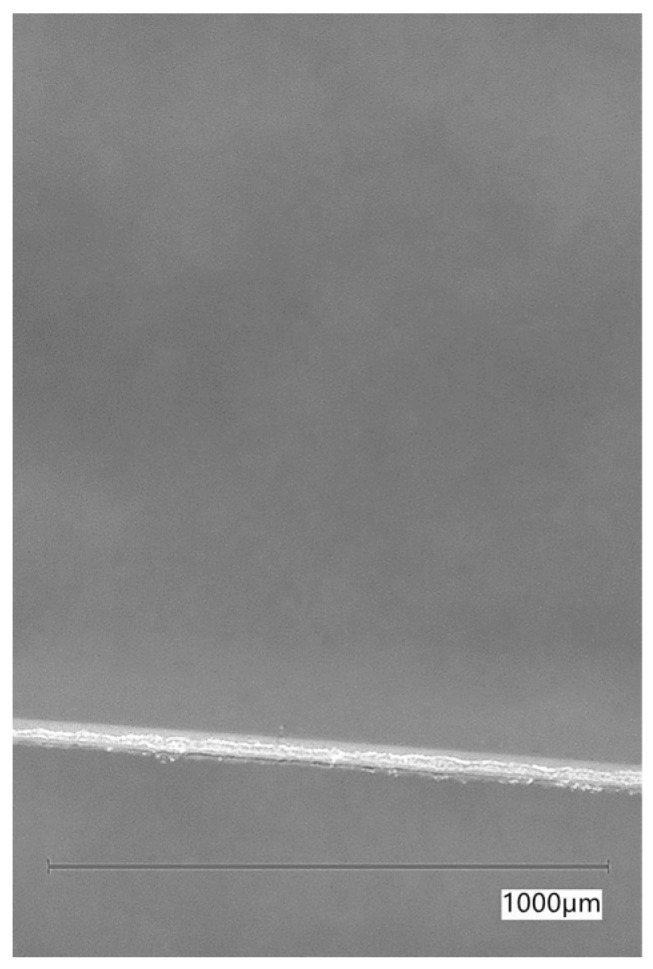
PE-LD 65×.

**Figure 10:**
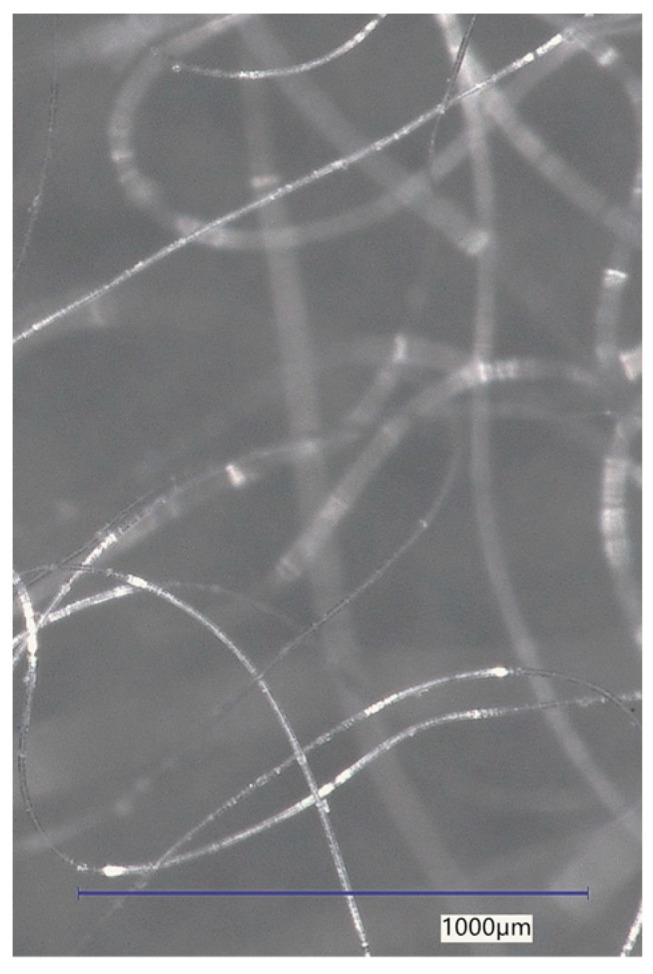
PES 65×.

The thickness, length, and shape of the chosen fibre types were determined using an optical microscope at various magnifications. The recording was conducted with a Keyence VHX digital optical microscope (Keyence Corporation, Osaka, Japan). The images of the optical microscope are shown in Figure 7, Figure 8, Figure 9 and Figure 10 at a magnification of 65. The properties determined using the optical microscope were observed only for PP, RE-PET, and PES.

As can be seen from the values listed in Table 1, PES and RE-PET fibres have similar thickness, while PP fibres are thicker. In the case of LD-PE, these were experimentally produced fibres, but in practice, their thicknesses are generally similar to those of PP fibres. In terms of fibre length, the longest were PES fibres, while the shortest were RE-PET fibres. However, the fibre length is not crucial in this case, since all polymer fibres are produced in various lengths and can be further modified if necessary.

#### 2.1.2. Scanning Electron Microscopy (SEM)

**Figure 11:**
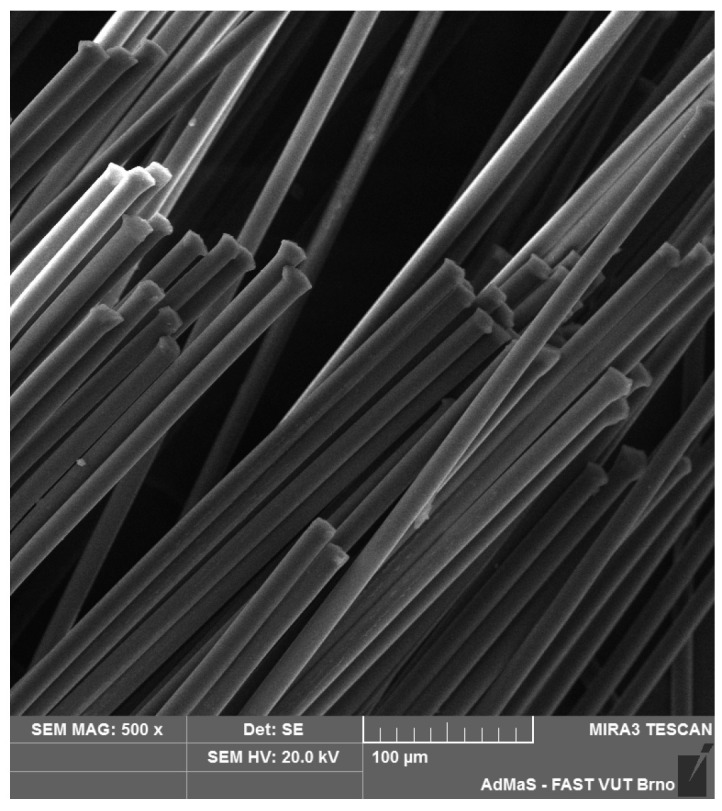
RE-PET 500×.

**Figure 12:**
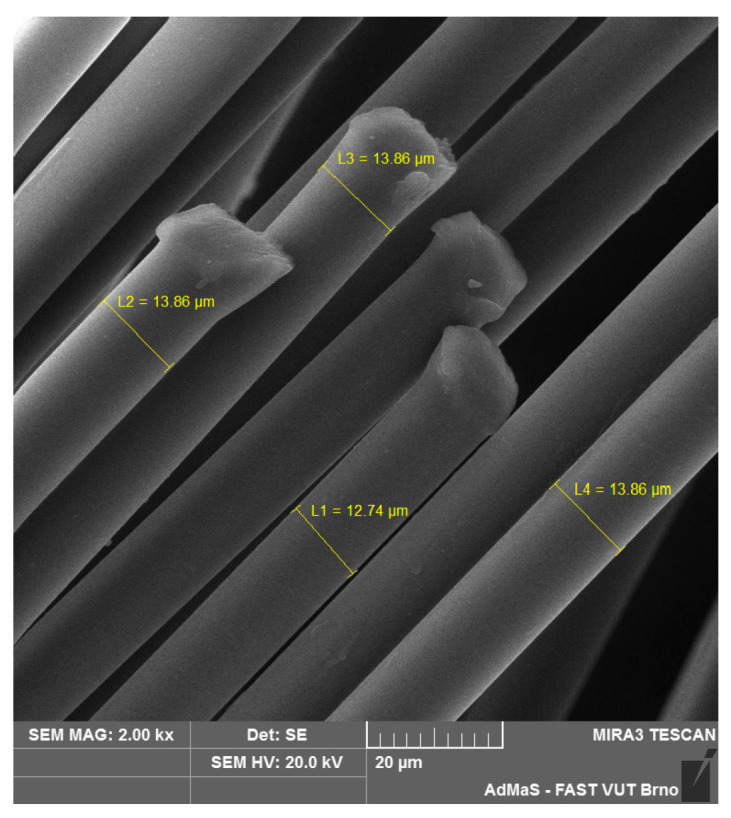
RE-PET 2000×.

**Figure 13:**
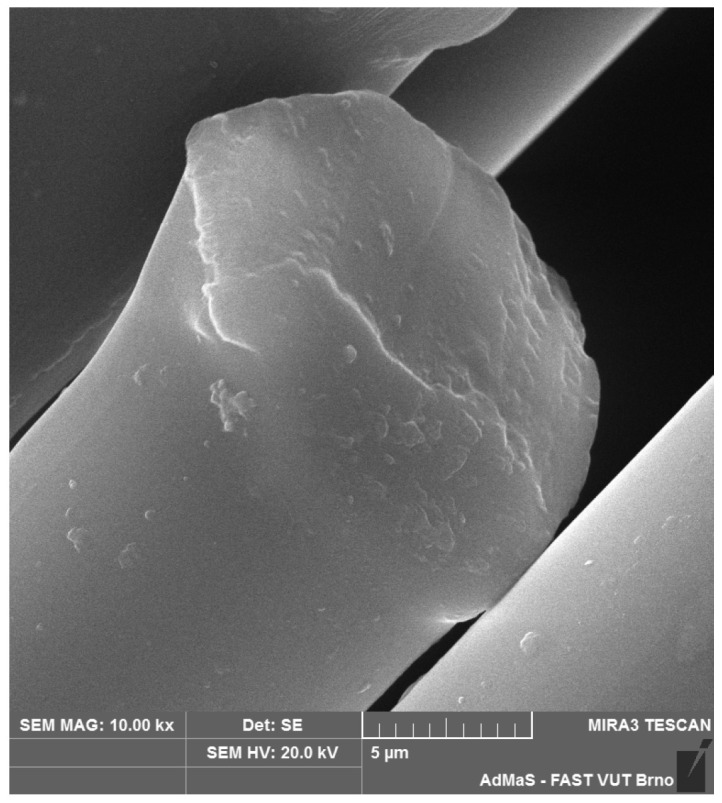
RE-PET 10,000×.

**Figure 14:**
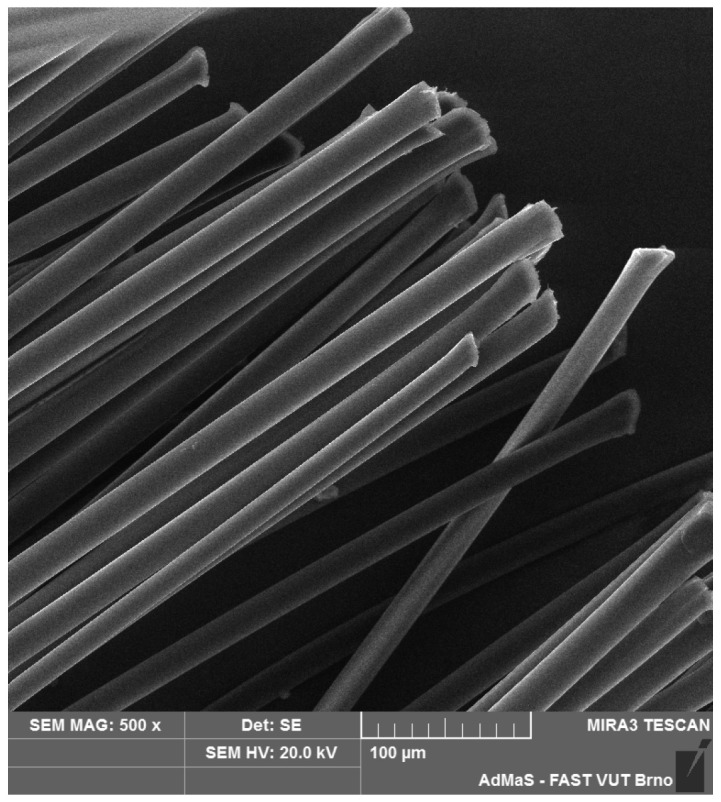
PP 500×.

**Figure 15:**
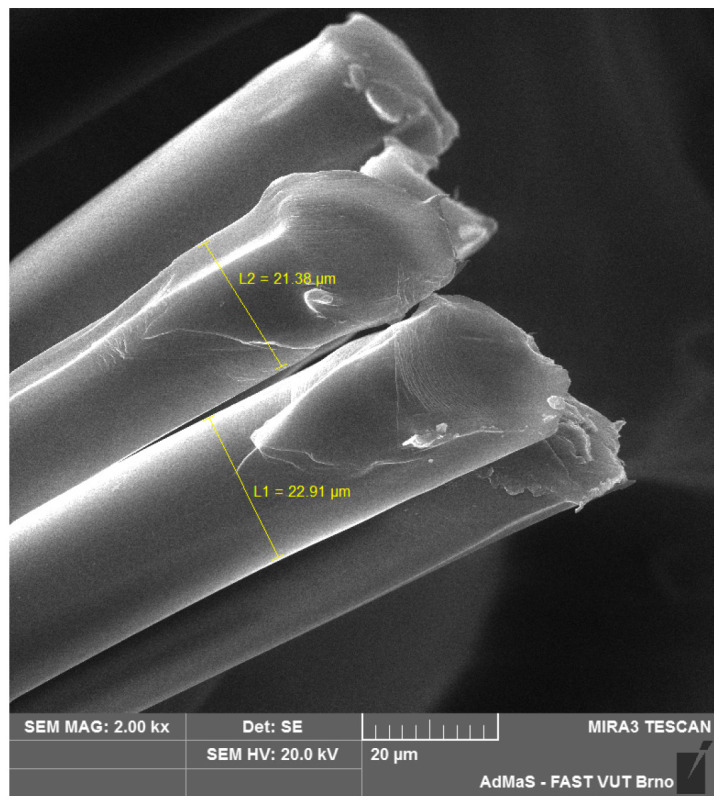
PP 2000×.

**Figure 16:**
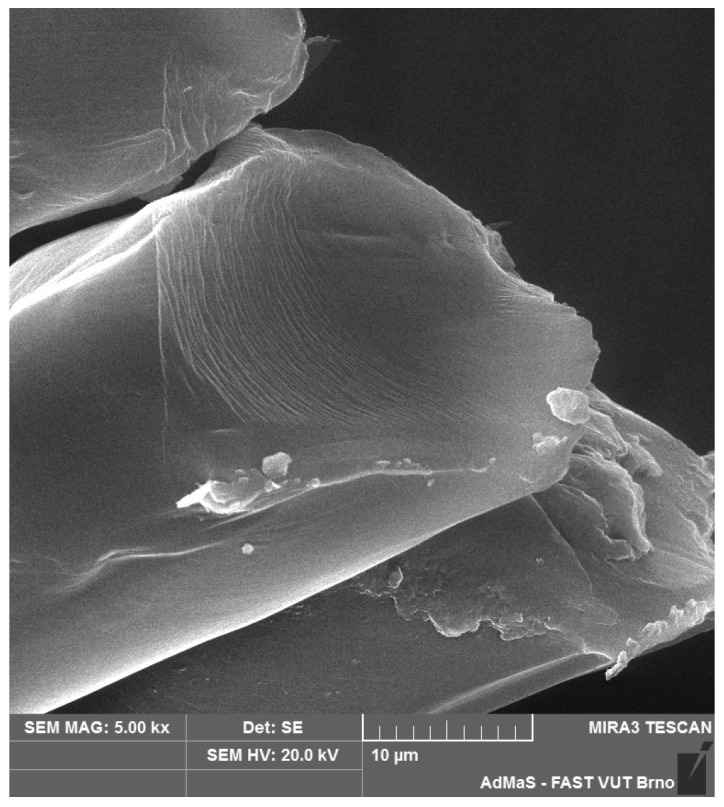
PP 5000×.

**Figure 17:**
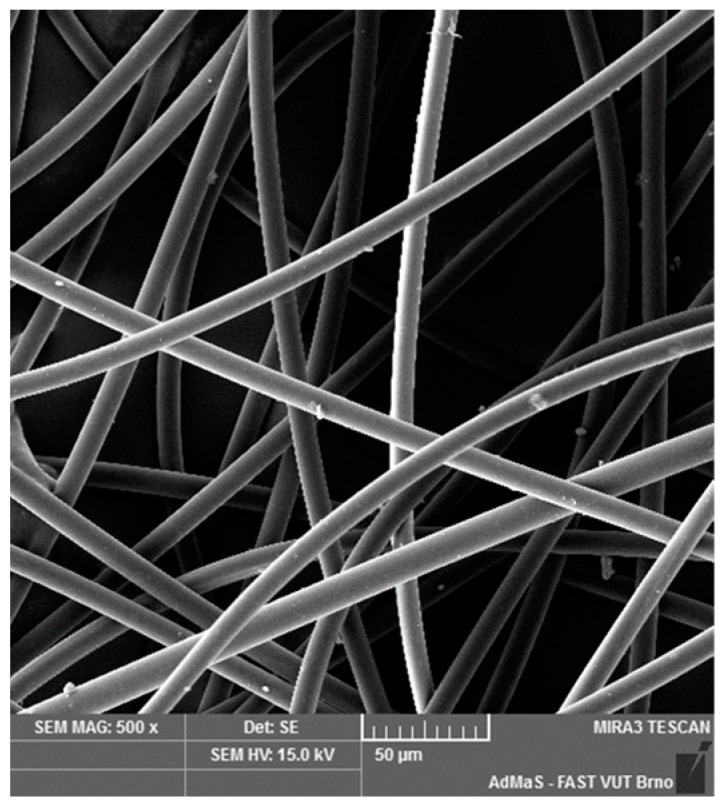
PES 500×.

**Figure 18:**
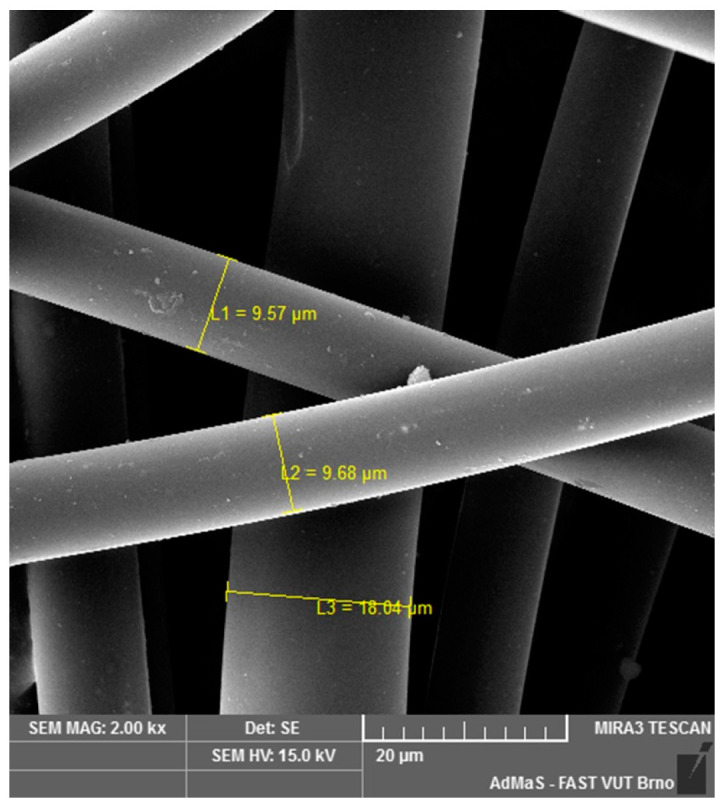
PES 2000×.

**Figure 19:**
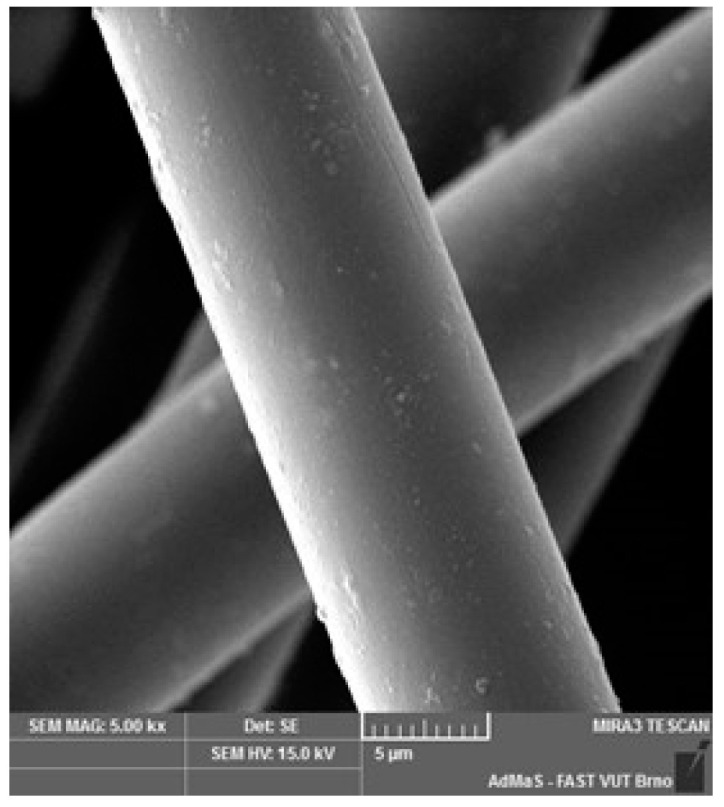
PES 5 10,000×.

SEM analysis was used in both sections of the experimental work. First, individual RE-PET, PP, and PES fibres were studied, including their cross section, shape, surface, and width, as shown in Figure 11, Figure 12, Figure 13, Figure 14, Figure 15, Figure 16, Figure 17, Figure 18 and Figure 19. Subsequently, SEM analysis was used to observe the behaviour of fibres in concrete, their anchoring in the cement matrix, to determine the presence of fibres after exposure to elevated temperatures and to assess the homogeneous distribution of fibres in concrete.

As can be seen from Figure 11, Figure 12, Figure 13, Figure 14, Figure 15, Figure 16, Figure 17, Figure 18 and Figure 19, all three fibre types have approximately similar properties, with the fibres having a regular cross-section. RE-PET fibres can be seen in various magnifications in Figure 11, Figure 12 and Figure 13, as can PP fibres in Figure 14 and Figure 15 and PES fibres in Figure 17, Figure 18 and Figure 19. The fibres have a circular cross section, and the surface of the fibres is smooth without deformation. The only deformation of the fibres is at the end of the fibre, which is caused by cutting/shortening of long fibres to the desired length. The results confirm the findings obtained from the optical microscopy described above.

#### 2.1.3. Thermal Analysis

**Figure 20:**
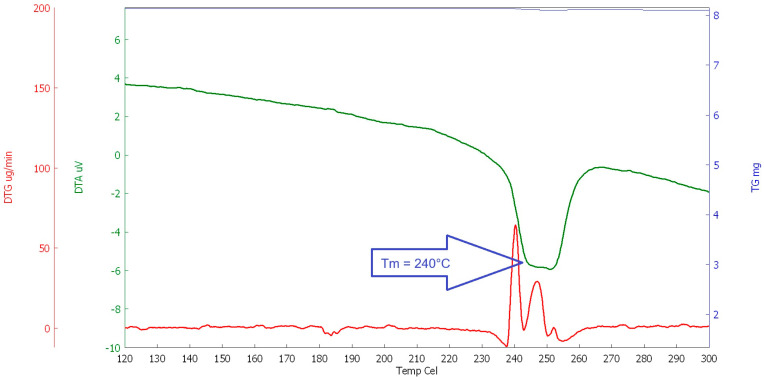
DSC RE-PET.

**Figure 21:**
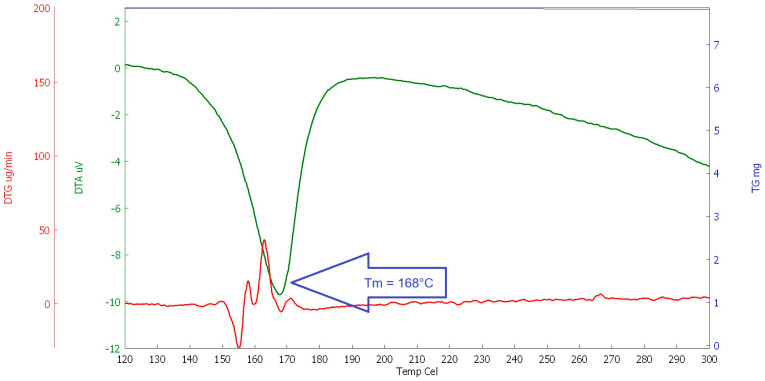
DSC PP.

**Figure 22:**
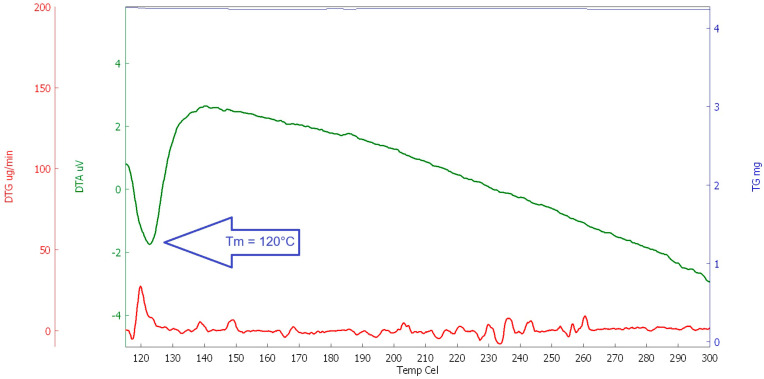
DSC PE_LD.

**Figure 23:**
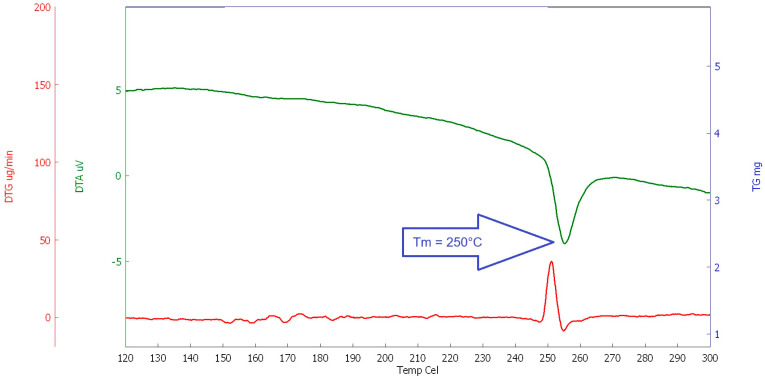
DSC PES.

For the study of the behaviour of selected polymers at elevated temperatures, a thermal analysis (DSC, DTA, and TG) was performed. The heating rate was set at 10 °C/min (in the temperature range of 23 to 600 °C, the interval between 100 and 300 °C was evaluated for the determination of the melting point, see Figure 20, Figure 21, Figure 22 and Figure 23). This analysis was supplemented by optical scanning of the samples during heating to visually assess changes in the condition and structure of the fibres.

The main goal was to evaluate the melting point of the fibres, followed by their decomposition and burnout (depending on the type of fibre). The evaluation was carried out comprehensively, taking into account all measured values for each type of analysis.

As can be seen in the above graphs, PE-LD has the lowest melting temperature, melting at approximately 120 °C, followed by PP, which melts at around 168 °C, RE-PET at around 240 °C, and finally pure primary PES melts at around 250° C. In this case, it can be concluded that both RE-PET and PES melt at relatively high temperatures and are less suitable for use in concrete. However, even for these fibres, it can be expected that they will function in certain types of concrete during fires.

#### 2.1.4. Determination of the Gross Heat of Combustion

The test was carried out according to the EN ISO 1716 standard [39]. The value was determined using a semiautomatic IKA C 200 device (IKA-Werke GmbH & Co. KG, Staufen im Breisgau, Germany) under standard laboratory conditions. A prescribed amount of fibre samples was taken to determine five heat of combustion values from one measurement. The result is the average of three measurements of the heat of combustion for each sample. The results are presented in Table 2.

### 2.2. Properties of Concretes with Polymer Fibres

- Consistency of fresh concrete according to [40];
- Air content in fresh concrete according to [41];
- Bulk density in fresh and hardened states according to [42,43];
- Compressive strength after 28 days and residual compressive strength after exposure of samples to high temperatures according to [44].

As part of the test of the influence of polymer fibres on concrete properties, test mix designs were developed. Seven test formulations were proposed, using different amounts of PP and RE-PET fibres, with the same amount of water dosed, and the consistency was adjusted using a superplasticizer (based on polycarboxylate from the Mapei, spol. s r.o., Olomouc, Czech Republic). Polymer fibre concentration was chosen according to the recommendation of the PP fibre producers and according to previous own work with PP and RE-PET fibres:

- 660 g/m^3^—standard recommended concentration;
- 330 g/m^3^—50% of the recommended concentration;
- 999 g/m^3^—150% of the recommended concentration.

The proposed formulations are presented in Table 3.

The test samples were prepared in a standard manner in a forced circulation mixer. Fibres were dosed to the dry ingredients, which were homogenized before water was added. During the production of fresh concrete, the homogeneous distribution of the fibres was visually checked to avoid clumping or poor mixing of the fibres. Checks were also made on hardened concrete on the fracture surfaces of the samples after the mechanical properties were determined visually macroscopically and by optical microscopy. For both types of fibre, a uniform distribution of fibres in the volume of test specimens was achieved. When mixing the fibres, there is no problem with homogenisation throughout the concrete mix, the fibres are very quickly and homogeneously dispersed throughout the mix. The fibre distribution can be observed very well by eye in fresh and hardened concrete.

#### 2.2.1. Determination of the Consistency of Fresh Concrete

**Figure 24:**
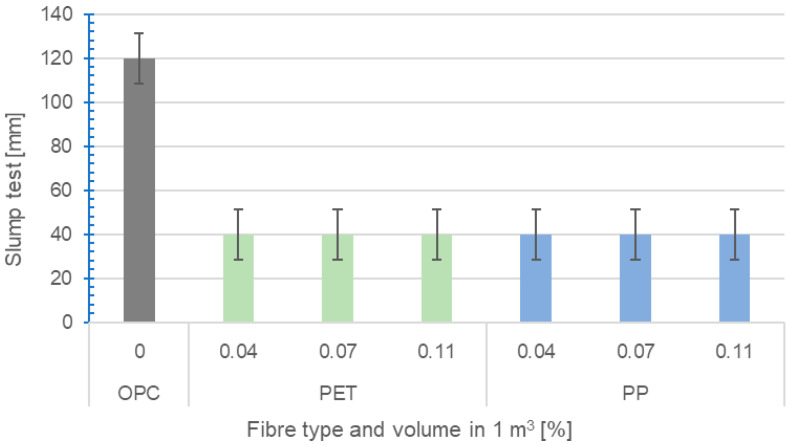
Determination of the effect of fibres on the consistency of concrete.

The determination of consistency was carried out using a slump test method according to EN 12350-2 [40]. This method was chosen due to its clear demonstration of the negative effect of adding any type of fibre on the workability of concrete. The results are shown in Figure 24 below.

The reference OPC mix was designed as pumpable concrete with a slump of 120 mm (S3). After adding PP or RE-PET fibres, there was a significant reduction in workability (slump of 40 mm). The reduction in workability was due to the increased surface area of the fibres, which needed to be coated with water. The water is then no longer available to ensure the desired workability of the concrete mix. To achieve the required consistency, this water must be replaced with an adequate amount of superplasticizer.

#### 2.2.2. Determination of Bulk Densities and Air Content in Fresh Concrete

**Figure 25:**
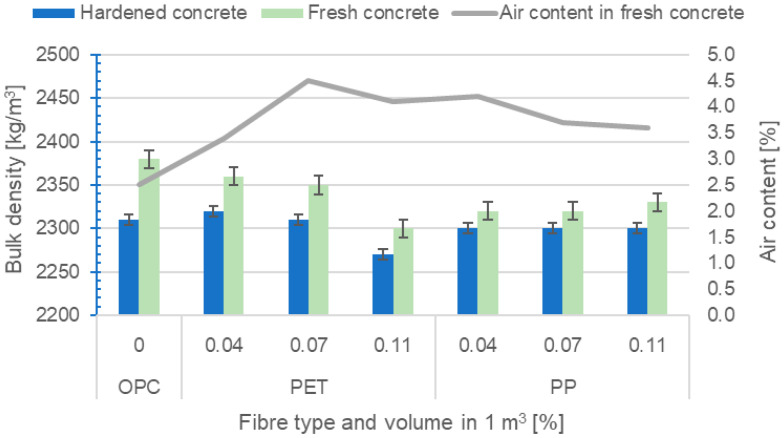
Bulk densities and air content in concrete.

The bulk density in the fresh state was determined according to EN 12350-6 [42] and in the hardened state according to EN 12390-7 [43]. The air content in the fresh concrete was determined according to EN 12350-7 [41] using the pressure method. The results of the bulk densities and air content in the concrete are presented in Figure 25.

The bulk density of fresh concrete ranges between 2300 and 2380 kg/m^3^, and for hardened concrete, it ranges between 2270 and 2310 kg/m^3^. The mix without fibres has a higher bulk density than most fibre-reinforced mixes and also has the lowest air content in fresh concrete. In the case of fibre-reinforced mixes, the effect of the fibre type is noticeable, whereas in the case of PP fibre mixes, the effect of the fibre dose on the resulting bulk density is not noticeable. In the case of RE-PET fibres, which have a significantly higher fineness and a shorter length, this effect is, in contrast, noticeable, since the number of active fibres increases more significantly with increasing fibre weight compared to that of PP fibres. Based on the measurements, it is evident that the use of fibres increases the air content in fresh concrete, which can lead to slight lightening and, consequently, to a reduction in bulk density. Thus, the addition of RE-PET fibres causes the greatest aeration, and therefore, the bulk density of concretes made with RE-PET fibres is most affected by the fibre dosage. However, in general, these are very small differences in terms of the bulk density of the concrete.

#### 2.2.3. Determination of Compressive Strength

**Figure 26:**
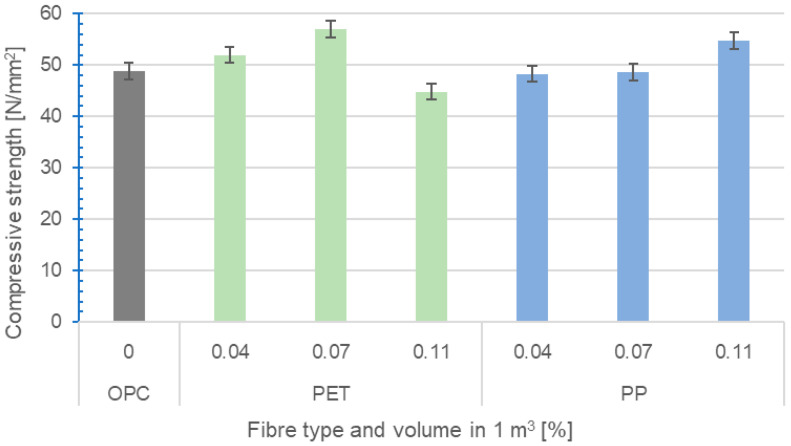
Compressive strength.

The compressive strength of the concrete was determined according to EN 12390-3 [44]. Cube test specimens with an edge length of 150 mm were tested at 28 days on a hydraulic press. The results of the compressive strengths are presented in Figure 26 below.

The compressive strengths corresponded relatively well with the bulk densities. For RE-PET 0.07 and PP 0.11, there was an increase in compressive strengths of 17% for RE-PET and 12% for PP. In the specimens with PP fibres, the addition of 0.04–0.11% fibres slightly improved the mechanical properties, which is mainly due to the reduction in micro-cracks in the initial stage of hydration and more efficient distribution of the stresses in the concrete [45,46]. For the samples with RE-PET fibres, this effect was only noticeable up to a dose of 0.07%; at a dose of 0.11%, the amount of fibres was already too large, which, together with the lower bulk density, led to a loss of mechanical properties.

**Figure 27:**
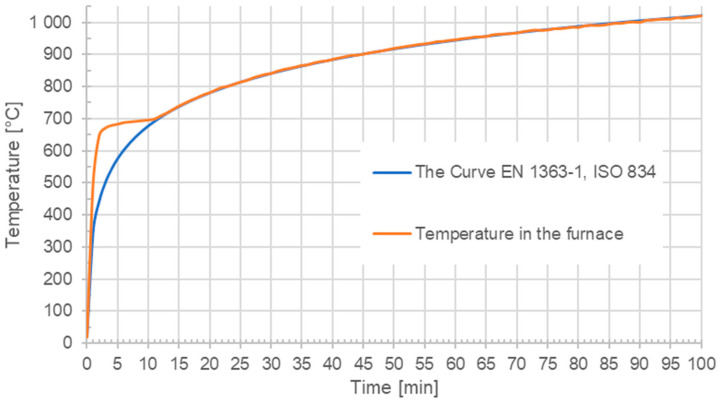
Fire curve according [45,46].

The next step of the experiment involved exposing the test specimens to extremely high temperatures. A new set of 150 mm edge length cube test samples was prepared, which was then placed in a fire oven after 28 days of mixing and subjected to a fire curve according to EN 1363-1 [47] and ISO 834 [48] (Figure 27). Subsequently, after cooling, the test specimens were removed from the furnace, and the residual compressive strength was determined.

**Figure 28:**
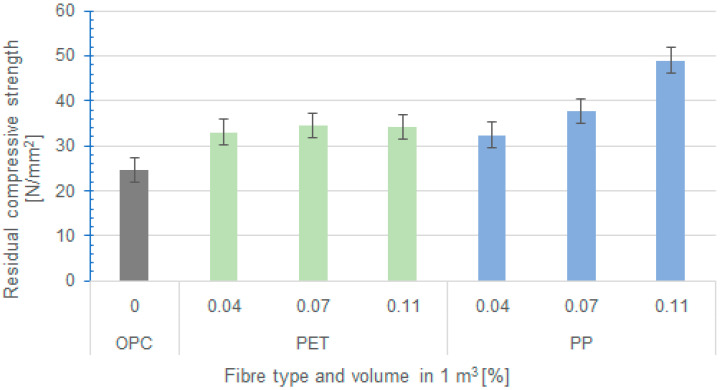
Residual compressive strengths after exposure to the fire curve.

The results of the residual compressive strengths are presented in Figure 28.

From the measured results, there is a clear positive influence of the fibres on ensuring the residual compressive strength, which is lowest in the reference concrete (OPC). However, in this case, it can be seen that two effects are included in the measured values that affect the resulting residual strengths. These are the initial strength of the concrete and the change in strength after exposure to the fire curve. In the case of PP fibres, it can be seen that increasing the fibre dosage from 0.04 to 0.11% improves compressive strength and further reduces loss of mechanical properties after exposure to extreme temperatures. Therefore, the residual strengths increase for concretes with PP fibres with increasing fibre dosage. However, the situation is different for concretes with RE-PET fibres. As mentioned above, the addition of RE-PET fibres improves the mechanical properties only up to a dose of 0.07%. On the other hand, concrete with 0.11% fibre has a reduction in strength. In terms of reducing the drop in strength after thermal loading, these fibres act favourably with increasing concentration. Therefore, when both these effects are included together, the residual strengths of the samples with RE-PET fibres do not change significantly with the dose of fibre.

For better clarity, the evaluation is also presented using the percentage of strength retention compared to test samples that were not subjected to high temperatures, as shown in Table 4.

The provided Table shows the positive contribution of fibres in the fire protection area, as the compressive strength of the reference concrete without fibres decreased by almost 50%, compared to the concrete with fibres, where the decrease ranged from 60% to 90% depending on the type and amount of fibre. When comparing PP and RE-PET, the PP fibres exhibited a higher residual compressive strength.

**Figure 29:**
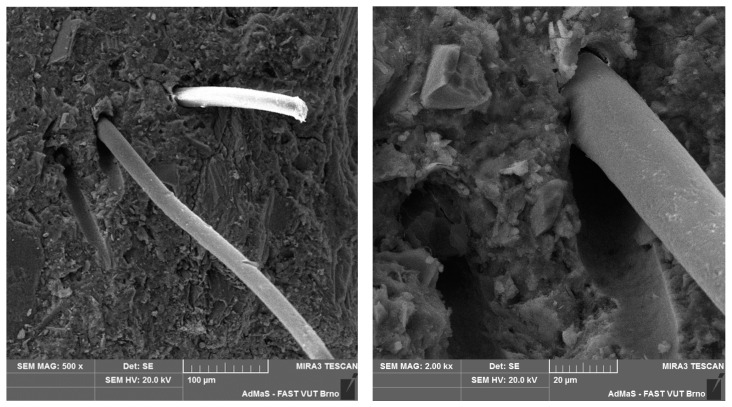
RE-PET in concrete 500× (**left**) and 2000× (**right**) magnification.

**Figure 30:**
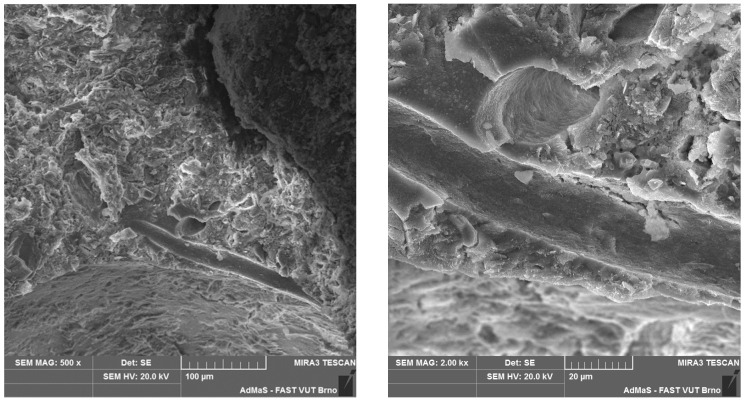
Air channels after the burnout of RE-PET fibres at 500× (**left**) and 2000× (**right**) magnification.

The SEM analysis was conducted on concrete fragments with RE-PET fibres after the compressive strength test for test samples both without and with exposure to high temperatures. The aim was to evaluate the anchoring of the fibres in the cement matrix and the formation of internal channels due to the burnout of the fibres as a result of high temperature. Observations recorded using SEM are presented in Figure 29 and Figure 30. As can be seen in the images, the thermal loading caused the fibres to burn out, while the structure of the composite remained intact without cracks.

## 3. Results and Discussion

In the scientific work conducted, four types of polymer fibres were selected to verify their potential utilization in the production of fire-resistant concrete. The following types of fibres were chosen: low-density polyethylene (LD-PE), polypropylene (PP), recycled polyethylene terephthalate (RE-PET), and polyester (PES) fibres. Their basic properties were determined, revealing that industrial fibres have a thickness ranging from 12 to 23 microns, while artificially prepared LD-PE fibres had a thickness ranging from 38 to 45 microns. The length of the fibres ranged from 6 to 32 mm. The melting temperature and combustion heat of the fibres were evaluated, with the LD-PE and PP fibres having a melting temperature lower than 170 °C, making them more suitable for use in concrete than the RE-PET/PES fibres. However, LD-PE/PP fibres have approximately twice the combustion heat compared to RE-PET/PES fibres, suggesting that from the perspective of fibre burnout, RE-PET/PES fibres are more suitable than LD-PE/PP fibres.

For further work, PP and RE-PET fibres were chosen to determine whether one type of fibre is truly advantageous or whether the fibres are comparable in practical use. According to available studies [28,29,30,31,32,33,34,35,36,37], PP fibres were expected to perform better; however, a partially positive effect of PET fibres was also confirmed. Concrete formulations with different contents of RE-PET and PP fibres were proposed. In preparation and on hardened samples, it was verified that a uniform mixing and distribution of the fibres occurred.

From a rheological perspective, the influence of both types of fibres on the behaviour of fresh concrete was found to be approximately comparable (within the doses used of 0.04, 0.07, and 0.11%). The use of fibres affected the bulk densities of both fresh and hardened concrete. For RE-PET fibres, the bulk density decreased along with the dose of fibre, while for PP, the bulk density remained approximately constant.

The addition of RE-PET fibres causes more aeration compared to PP, and therefore, the bulk density of concretes made with RE-PET fibres is most affected by the fibre dosage. This is due to the higher fineness and shorter length of RE-PET fibres compared to PP. At the same weight rate, the number of active RE-PET fibres is significantly higher than the number of PP fibres.

In terms of compressive strength in hardened concrete, an improvement in mechanical properties was observed for most formulations, except for the RE-PET 0.11 formulation, where the compressive strength decreased below that of fibre-free concrete.

Here, it was found that in the case of RE-PET fibres, a double effect occurs, where the fibres improve the structure of the resulting composite and prevent the formation of microcracks in the initial stage of hydration but cause more aeration at higher additions. Therefore, the addition of 0.11% RE-PET fibres results in a decrease in mechanical properties.

After exposure of the concrete to high temperatures according to EN 1363-1 and ISO 834, it was found that the residual strengths of all formulations were higher than those of fibre-free concrete, with the residual strengths of samples containing PP fibres in doses of 0.07% and 0.11% higher than those of the other samples. Despite these findings, it can be stated that a positive effect was significantly demonstrated for all fibres.

In concretes with RE-PET fibres, there is a noticeable effect of fibre overdose (described above) and a higher level of aeration; therefore, increasing the fibre dosage from 0.07 to 0.11% does not improve residual strength any more because although the damage rate of the concrete is reduced, its mechanical properties before thermal loading decrease.

## 4. Conclusions

In the experiments conducted, it was found that the addition of polymer fibres has a positive effect on the properties of concrete in the hardened state and can significantly improve its behaviour when exposed to high temperatures during a fire. Overall, it was determined, according to the initial assumption, that PP fibres are better for the given application, and because of their lower melting temperature, they are more suitable for improving fire properties than PET or PES fibres. However, at doses of 0.04 and 0.07, a demonstrable improvement in mechanical properties (compressive strength) was observed in hardened concrete, and these fibres enhanced the functional properties of hardened concrete, unlike PP fibres, which only showed improved mechanical properties at the highest dose of 1%. In this case, however, the different thicknesses and lengths of the fibres must be taken into account. PP fibres have a thickness of 20–23 microns and a length of 12.1 mm. RE-PET fibres have a thickness of only 13–15 microns and a length of approximately 6 mm. Therefore, for the same-weight batch, the number of RE-PET fibres is about 5 times greater than the number of PP fibres. Therefore, at higher doses, RE-PET fibres start to negatively affect the key properties, as they significantly increase the aeration of the concrete and start to degrade the mechanical properties.

In terms of residual strengths, the best properties were exhibited by the 0.11% PP fibre mix, but the three variants with RE-PET fibres also showed a demonstrable improvement in residual strengths compared to the control mix without fibres. In general, it can be stated that formulations with 0.04 and 0.07% RE-PET fibres exhibited better properties than the control formulation without fibres, both before and after exposure to high temperatures. Although the properties were not as favourable as those with the addition of 0.11% PP fibres, the results can be considered very positive, and RE-PET fibres can be used successfully in concrete for this purpose. Because these are recycled fibres, this application is more interesting and environmentally economical from an LCA perspective compared to the use of virgin PP-based fibres. As can be seen from the comparison of the compressive strengths of concrete without fibres and concrete with RE-PET fibres, the strength characteristics of the concrete can be improved by an appropriate dose of fibres (in this case, 0.07%), and it would be possible to reduce the cement dose in this case while maintaining comparable mechanical properties with concrete without fibres. In this way, further economic and environmental savings can be achieved, and good concrete properties can be ensured after exposure to high temperatures.

## Figures and Tables

**Table 1:** Properties of fibres determined by the optical microscope.

Fibre Type/Property	PP	RE-PET	LD-PE	PES
Thickness [µm]	20–23	13–15	38–45	12–16
Face	straight	straight	straight	straight
Surface	smooth	smooth	smooth	smooth
Length [mm] ^1^	12.12	5.98	14.25	32.15

^1^ Length and thickness were measured on 20 randomly selected fibres, and the average was determined.

**Table 2:** Values of the heat of combustion of polymer materials.

Sample Designation	RE-PET	PP	PE-LD	PES
Q_PCS_ [MJ/kg]	23.09	45.22	45.81	21.28

**Table 3:** Concrete mix designs tested with fibres, composition per 1 m^3^.

Unit	OPC ^1^	MA 0/4 ^2^	MA 4/8 ^2^	CA 8/16 ^3^	W ^4^	P ^5^	F ^6^
	kg						
OPC	350	945	315	600	180	0.8	0.00
RE-PET 0.04	350	945	315	600	180	1.0	0.50
RE-PET 0.07	350	945	315	600	180	1.3	1.00
RE-PET 0.11	350	945	315	600	180	1.5	1.50
PP 0.04	350	945	315	600	180	0.9	0.33
PP 0.07	350	945	315	600	180	1.0	0.66
PP 0.11	350	945	315	600	180	1.3	0.99

^1^ OPC (CEM I 42.5 R), ^2^ MA 0/4 (4/8)—mined aggregate fraction 0/4 (4/8) mm, ^3^ CA 8/16—crushed aggregate fraction 8/16 mm, ^4^ W—water, ^5^ P—superplasticizer, ^6^ F—fibres (the mix designation indicates the type of fibre and its amount per 1 m^3^ in %)—variable parameters (column P—amount of plasticizer and F fibres) are marked in this Table.

**Table 4:** Residual compressive strength [%] after exposure to the fire curve.

OPC	RE-PET 0.04	RE-PET 0.07	RE-PET 0.11	PP 0.04	PP 0.07	PP 0.11
50.5	63.6	60.6	76.4	67.1	77.7	89.5

## Data Availability

The original contributions presented in the study are included in the article, further inquiries can be directed to the main authors.
